# AGE BIAS IN ARTIFICIAL INTELLIGENCE (AI): A VISUAL PROPERTIES ANALYSIS OF AI IMAGES OF OLDER VERSUS YOUNGER PEOPLE

**DOI:** 10.1093/geroni/igad104.3168

**Published:** 2023-12-21

**Authors:** Laura Allen, Wenqian Xu, Mariko Nishikitani, Vaishnavi Atul Patil, Dana Bradley

**Affiliations:** University of Maryland, Baltimore County, Baltimore, Maryland, United States; Lund University, Lund, Skane Lan, Sweden; Kyushu University Hospital, Fukuoka, Fukuoka, Japan; University of Maryland, Baltimore County, Baltimore, Maryland, United States; University of Maryland, Baltimore County, Baltimore, Maryland, United States

## Abstract

Concerns about age-related biases in Artificial Intelligence (AI)’s design, development, and data generation raise alarms for potential social inequalities. AI art-generators like MidJourney, known for creating distinctive and artistic images, have gained attention recently. However, the critical examination of their potential biases remains limited. Our study aims to investigate age-related bias in MidJourney by comparing how older and younger individuals are visually depicted. Using 19 keywords (as text prompts) tied to domains of potential social exclusion for older adults, we automated the collection of “old person” and “young person” images in MidJourney. The text-to-image prompts yielded 456 AI-generated images that were analyzed with Amazon Rekognition image detection software to transform them to quantitative data. Data were compared statistically using STATA (version 16). The results show that the images of older people are significantly less bright (p< 0.001) and less sharp (p< 0.001) than the images of younger people. Additionally, images of older people have significantly more smile expressions (16%; p< 0.001) and are more likely to be wearing glasses (56%; p< 0.001) compared to images of younger people (6% and 12%, respectively). The findings reveal that MidJourney’s algorithm associates older people with a negative sense of antiquity, irrelevance, and dread. The findings also align with the Stereotype Content Model in which older people are seen as having warm personalities but not as competent or capable as younger people. We interpret these results as age bias that the AI art generator MidJourney has learned and in turn, creates, through image generation.

